# Association between Coffee Consumption and Brain MRI Parameters in the Hamburg City Health Study

**DOI:** 10.3390/nu15030674

**Published:** 2023-01-28

**Authors:** Carola Mayer, Felix L. Nägele, Marvin Petersen, Maximilian Schell, Ghazal Aarabi, Thomas Beikler, Katrin Borof, Benedikt M. Frey, Julius Nikorowitsch, Juliana Senftinger, Carolin Walther, Jan-Per Wenzel, Birgit-Christiane Zyriax, Bastian Cheng, Götz Thomalla

**Affiliations:** 1 Department of Neurology, University Medical Center Hamburg-Eppendorf, 20246 Hamburg, Germany; 2 Department of Periodontics, Preventive and Restorative Dentistry, University Medical Center Hamburg-Eppendorf, 20246 Hamburg, Germany; 3 Department of Cardiology, University Heart and Vascular Center, 20246 Hamburg, Germany; 4 Midwifery Science—Health Service Research and Prevention, Institute for Health Services Research in Dermatology and Nursing (IVDP), University Medical Center Hamburg-Eppendorf, 20246 Hamburg, Germany

**Keywords:** cerebral small vessel disease, coffee consumption, cortical atrophy, diffusion-weighted magnetic resonance imaging, microstructural integrity, neurodegenerative diseases, white matter hyperintensities

## Abstract

Despite associations of regular coffee consumption with fewer neurodegenerative disorders, its association with microstructural brain alterations is unclear. To address this, we examined the association of coffee consumption with brain MRI parameters representing vascular brain damage, neurodegeneration, and microstructural integrity in 2316 participants in the population-based Hamburg City Health Study. Cortical thickness and white matter hyperintensity (WMH) load were measured on FLAIR and T1-weighted images. Microstructural white matter integrity was quantified as peak width of skeletonized mean diffusivity (PSMD) on diffusion-weighted MRI. Daily coffee consumption was assessed in five groups (<1 cup, 1–2 cups, 3–4 cups, 5–6 cups, >6 cups). In multiple linear regressions, we examined the association between brain MRI parameters and coffee consumption (reference group <1 cup). After adjustment for covariates, 3–4 cups of daily coffee were associated with lower PSMD (*p* = 0.028) and higher cortical thickness (*p* = 0.015) compared to <1 cup. Moreover, 1–2 cups per day was also associated with lower PSMD (*p* = 0.022). Associations with WMH load or other groups of coffee consumption were not significant (*p* > 0.05). The findings indicate that regular coffee consumption is positively associated with microstructural white matter integrity and cortical thickness. Further research is necessary to determine longitudinal effects of coffee on brain microstructure.

## 1. Introduction

Coffee is one of the most consumed beverages worldwide. Even subtle effects of coffee on health might have wide-ranging implications on the population level [1]. Since coffee constituents such as caffeine can easily cross the blood-brain barrier, coffee is suggested to impact neurological health [2]. For example, regular coffee consumption was associated with a lower risk of cerebrovascular and neurodegenerative disease such as stroke, Parkinson’s disease, dementia, and cognitive decline [3,4,5,6,7,8]. These neuroprotective associations can be explained by the various ingredients of coffee which have anti-inflammatory properties, reduce amyloid-β levels in the cells, and decrease cardiovascular risk [9,10,11,12].

Despite the association of regular coffee consumption with fewer neurodegenerative diseases, it remains unclear how coffee is associated with pre-clinical brain pathologies such as lesions in the white matter, degeneration of the cortex, or alterations of the microstructural integrity. White matter hyperintensities (WMH) are hyperintense lesions on T2-weighted images and are associated with an increased risk for stroke and depression, cognitive deterioration, and gait disorders [13,14,15]. As a marker of cerebral small vessel disease (CSVD) and vascular brain damage, WMH can vary in the degree of expression, depending on the age and the presence of cardiovascular risk factors, e.g., smoking or hypertension [16,17,18]. Previous studies have reported diverging results on the association of consumed coffee with imaging markers of CSVD. They found either beneficial associations of coffee with lacunar infarcts [7], beneficial [19] or detrimental [20] associations with WMH volume, or no significant associations at all [21,22].

A recently developed and valid imaging marker of microstructural integrity is the peak width of skeletonized mean diffusivity (PSMD), calculated as the distribution of the mean diffusivity (MD) between the 5th and 95th percentile in the white matter skeleton [23]. Only one study analyzed the association of coffee consumption with microstructural integrity, as quantified by fractional anisotropy, with a higher coffee consumption being associated with higher integrity of the white matter microstructure [24].

Damage to the brain structure is not restricted to white matter, but also extents to the cortex, e.g., in the form of atrophy. Except for one study focusing on the quantification of cortical thickness in regions susceptible for Alzheimer’s Disease [22], the link between coffee consumption and cortical thickness was only indirectly examined by measuring total brain volume or grey matter volume, with incongruent results between studies [7,21,25,26]. This study aimed at investigating whether coffee consumption is associated with multiple brain MRI markers of vascular brain damage and neurodegeneration, including WMH, PSMD, and cortical thickness in a large, population-based cohort.

## 2. Materials and Methods

### 2.1. Study Design

This study included data from the Hamburg City Health Study (HCHS), an ongoing population-based, single-center, prospective cohort study investigating risk factors of major chronic diseases to improve prognosis and treatment options. A detailed description of the study design was published previously [27]. To describe briefly, 45,000 citizens of the city of Hamburg, Germany, between the age of 45 and 74 years are invited to receive an extensive baseline clinical evaluation. An MRI of the brain is conducted in a subgroup which comprises participants with increased cardiovascular diseases risk (determined by a Framingham Risk Score >7) and control participants [28]. This study included data from the first 10,000 participants, of which 2652 received a brain MRI. A total of 17 participants were excluded because of at least one missing MR sequence (12 without FLAIR, 14 without T1, 14 without diffusion-weighted MRI), 26 participants had to be excluded because of anomalies in the neuroradiological evaluation, 29 participants had to be excluded because of insufficient FLAIR (N = 23) or T1-weighted (N = 6) image quality, and 80 participants had to be excluded because of technical issues during brain parcellation (N = 53) or WMH segmentation (N = 27). Of the remaining participants, 2316 had complete data on coffee consumption.

### 2.2. Clinical Data Assessment

Coffee consumption was quantified as the average number of cups consumed per day over the last 12 months, based on the participants’ responses on a validated food frequency questionnaire (FFQ) [29]. The FFQ also examined the regular consumption of decaffeinated coffee. The questions were stated as follows: “Over the last 12 months, how often did you consume caffeinated/decaffeinated coffee? This also includes the consumption of espresso, cappuccino, café latte, and other preparation types.” One coffee corresponds to 150 milliliters. The frequency of coffee consumption was classified into five groups: less than one cup per day (<1 c/d), one to two cups per day (1–2 c/d), three to four cups per day (3–4 c/d), five to six cups per day (5–6 c/d), or more than six cups per day (>6 c/d). In addition, all participants received detailed anamnestic and clinical examination. For the current analysis, the covariates age, sex, educational status, diabetes, hypertension, smoking status, body mass index (BMI), adherence to the Mediterranean diet, and alcohol consumption were included. In short, educational status was evaluated on a scale from 1–3, based on the International Standard Classification of Education (ISCED) [30]. Diabetes mellitus was determined from self-reported prevalence, fasting serum glucose level (>126 mg/dL) or non-fasting serum glucose level (>200 mg/dL). Hypertension was determined based on self-reported prevalence, blood pressure (≥140/90 mmHg), or intake of antihypertensive medication. Smoking status was classified as active smokers or non-smokers. Adherence to the Mediterranean diet was calculated with the German version of the original Mediterranean Diet Adherence Screener (MEDAS) [31]. Alcohol consumption was measured as the frequency of alcohol consumption on a scale from 0–4, and converted into the monthly frequency.

### 2.3. MRI Acquisition and Processing

MR images were acquired on a single 3T Siemens Skyra MRI scanner (Siemens, Erlangen, Germany). All participants received the same imaging protocol. Structural imaging consisted of a 3D T1-weighted rapid acquisition gradient-echo sequence (MPRAGE; 256 axial slices, echo time (TE) = 2.12 ms, slice thickness (ST) = 0.94 mm, repetition time (TR) = 2500 ms, in-plane resolution (IPR) = 0.83 × 0.83 mm) and a 3D T2-weighted fluid-attenuated inversion recovery (FLAIR) image (192 axial slices, TR = 4700 ms, TE = 392 ms, 192 axial slices, ST = 0.9 mm, IPR = 0.75 × 0.75 mm). For single-shell diffusion MRI (dMRI), 75 axial slices were obtained covering the whole brain with gradients (b = 1000 s/mm^2^) applied along 64 noncollinear directions (TR = 8500 ms, TE = 75 ms, ST = 2 mm, IPR = 2 × 2 mm with anterior-posterior phase-encoding direction). MR data were preprocessed as described previously and full documentation of the MRI processing pipeline is available in a GitHub repository (https://github.com/csi-hamburg/CSIframe/wiki; accessed on 30 December 2022) [32]. In short, dMRI and T1-weighted images were preprocessed with QSIPrep 0.14.2. as implemented in Nipype 1.6.1. [33,34]. Among other steps, the pipeline includes intensity normalization, skull-stripping, MP-PCA denoising, removal of ringing artefacts, and correction for B1 field inhomogeneity, head motion, eddy current, and susceptibility distortions [34].

For the measurement of the brain volume and cortical thickness, brain parcellation was conducted on T1-weighted images using Freesurfer v.6.0.1. [35,36]. All images were thoroughly inspected both visually and quantitatively (outliers defined as measures exceeding 2 standard deviations from the median), and output of insufficient quality (i.e., segmentation errors) was excluded.

WMH were segmented as described in detail previously [32]. In short, we applied the Brain Intensity AbNormality Classification Algorithm (BIANCA) implemented in FSL with LOCally Adaptive Threshold Estimation (LOCATE) on preprocessed FLAIR images and T1-weighted images [37,38]. After applying both algorithms, the segmentations were refined using Freesurfer v.6.0.1 parcellations to exclude non-white matter regions (among others, corpus callosum and basal ganglia) [35]. Finally, the lesion load was calculated by normalization for intracranial volume, as calculated by Freesurfer v.6.0.1. [35]. The normalized lesion load is further referred to as ‘log WMH load’.

PSMD was calculated based on standard procedures and adapted for our purposes by using non-linear registration with the Advanced Normalization Tools (ANTs) SyN registration [23,39]. PSMD is calculated as the difference between the 95th and 5th percentile of MD values on the white matter skeleton in standard (MNI) space. White matter areas susceptible to partial volume effects of cerebrospinal fluid were excluded by masking.

### 2.4. Statistical Analyses

Demographical data were summarized and reported with median and interquartile range (IQR) for continuous variables and number and percentage (n, %) for categorical variables. Demographical characteristics and cardiovascular risk factors were tested for significant difference between groups of coffee consumption by applying Pearson Chi-square test for categorical variables and Kruskal-Wallis-test for continuous variables. To investigate the association between coffee consumption and MRI markers, we conducted multiple linear regression models with MRI markers as the dependent variables (mean cortical thickness, log WMH load, PSMD). Coffee consumption and covariates (age, sex, education, smoking status, diabetes, hypertension, BMI, adherence to Mediterranean diet, alcohol consumption) were added as independent variables to the models. The lowest group of coffee consumption (<1 cup per day) was set as the reference group. The output of the linear regression analysis was reported with standardized coefficients (β) and *p*-Values (*p*). A *p* < 0.05 was interpreted as significant. All statistical analyses were carried out using R software v.4.2.2. [40].

## 3. Results

### 3.1. Study Sample Characteristics

The final cohort consisted of data from N = 2316 participants who had a median age of 65 (IQR = 14) years, with 44.3% female participants. Of all participants included, 19.8% consumed <1 cup of coffee per day, 44% consumed 1–2 cups per day, 25.1% consumed 3–4 cups per day, 7.6% consumed 5–6 cups per day, and 3.5% consumed >6 cups of coffee per day. The consumption of decaffeinated coffee was less prevalent. In total, N = 127 (5.5%) reported consuming decaffeinated coffee on a regular basis (at least 1 cup per day). However, 120 of the 127 (94.5%) participants also drank caffeinated coffee, leaving only seven individuals exclusively consuming decaffeinated coffee. Since this group was too small for statistical analysis, we only included the consumption of caffeinated coffee.

**Figure 1:**
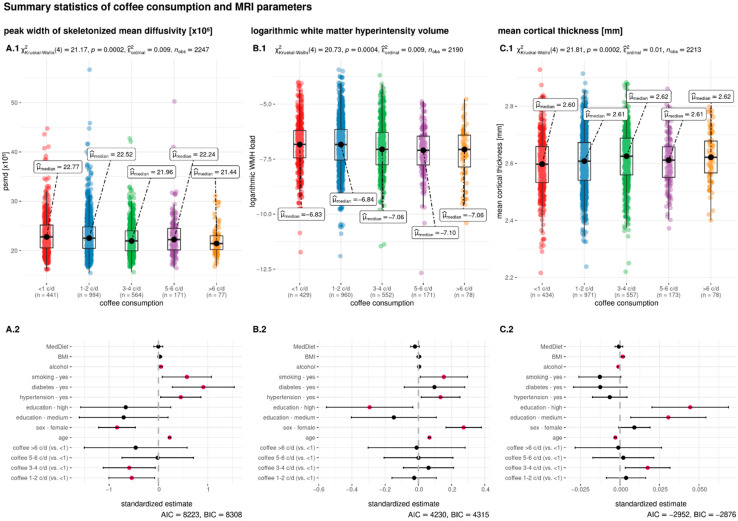
Summary statistics of the association between coffee consumption and the MRI parameters. Daily coffee consumption is indicated in cups per day (c/d) and categorized in five groups (<1 c/d, 1–2 c/d, 3–4 c/d, 5–6 c/d, >6 c/d). The upper row shows boxplots with the groups of coffee consumption on the x-axis and peak width of skeletonized mean diffusivity (PSMD, (**A.1**)) logarithmic white matter hyperintensity load (log WMH load, (**B.1**)) and mean cortical thickness (**C.1**) on the y-axis. The inferential test results above the boxplots indicate the output from the non-parametric Kruskal–Wallis test. The bottom row shows the results of the linear regression models where the dependent variable is either PSMD (**A.2**), log WMH load (**B.2**), or mean cortical thickness (**C.2**). Independent variables are the same in all regressions and presented on the y-axis. The standardized estimates are presented on the x-axis. Pink dots indicate a significant association and black dots a non-significant association of the independent variable. Abbreviations: AIC = Akaike information criterion; BIC = Bayesian information criterion; c/d = cups per day; MedDiet = adherence to the Mediterranean diet; mm = millimeter; n_obs_ = number of observations; *p* = *p*-Value; μ_median_ = median; χ^2^ = chi-square from the Kruskal–Wallis test.

Subjects consuming more caffeinated coffee were more often younger, male, of higher education, and possessing a higher BMI. Moreover, a higher coffee consumption was positively associated with smoking and negatively associated with diabetes mellitus and hypertension (Table 1). The median WMH volume was 1.48 mL (IQR = 2.22) and the median cortical thickness was 2.61 mm (IQR = 0.13). For a detailed description of the study sample, see Table 1. In Figure 1A.1,B.1,C.1, the distribution of the MRI parameters across the groups of coffee consumption are visualized.

### 3.2. Association of Coffee Consumption with MRI Parameters

After controlling for age, sex, level of education, diabetes mellitus, hypertension, smoking, BMI, Mediterranean diet, and alcohol consumption, the amount of coffee consumed was associated with PSMD and mean cortical thickness, but not with log WMH load (Table 2). A daily coffee consumption of <1 cup per day was set as the reference group. Drinking 1–2 cups of coffee per day was associated with lower PSMD, compared to the reference group (β = −0.542, *p* = 0.022). Moreover, 3–4 cups of coffee consumption per day was associated with lower PSMD, compared to group drinking <1 cup per day (β = −0.591, *p* = 0.028). Drinking 5–6 cups per day (β = −0.014, *p* = 0.97) or >6 cups per day (β = −0.461, *p* = 0.386) was not associated with a different PSMD, compared to the reference group. For cortical thickness, 3–4 cups of coffee were associated with significantly higher cortical thickness (β = 0.018, *p* = 0.015), compared to <1 cup per day. Drinking 1–2 cups of coffee per day was not significantly associated with a difference in cortical thickness (β = 0.004, *p* = 0.544) when compared to drinkers with <1 cup of daily consumption. The result was also insignificant for a daily consumption of 5–6 cups per day (β = 0.002, *p* = 0.831) and >6 cups per day (β = −0.001, *p* = 0.941). The association with log WMH load was not significantly different between the groups of coffee consumption, with <1 cup per day as the reference group (1–2 cups per day: β = −0.025, *p* = 0.71; 3–4 cups per day: β = 0.061, *p* = 0.429; 5–6 cups per day: β = 0.001, *p* = 0.994; >6 cups per day: β = −0.01, *p* = 0.944). See Table 2 for the detailed output of the linear regression.

## 4. Discussion

In this study, we examined the association of cortical thickness, cerebral small vessel disease (in the form of WMH), and microstructural integrity with coffee consumption in a population-based cohort including data from 2316 middle-aged to elderly participants. Our results show that individuals with a regular and moderate coffee consumption of 3 to 4 cups per day had higher cortical thickness and less microstructural white matter alteration, as quantified by the PSMD. Individuals drinking 5 or more cups per day did not significantly differ on any MRI parameter from individuals drinking <1 cup per day, indicating a U-shaped relationship. Moreover, there was no significant association of coffee with CSVD quantified by WMH, which is in congruence with the few studies examining the same association [7,21,22]. While PSMD measures subtle changes in the white matter microstructure and already captures early signs of structural damage, WMH reflects an end stage of white matter damage. Thus, our findings are novel in providing evidence for an association of a moderate coffee consumption with findings of early microstructural brain damage.

Multiple factors that are known to induce neurodegeneration and vascular brain damage can be beneficially influenced by the consumption of coffee, i.e., systemic inflammatory processes, deposition of amyloid-ß, and the development of cardiovascular risk factors. In the following section, we will discuss each factor in the context of coffee consumption and reflect these on the results of our analysis.

Of the more than 1000 bioactive compounds of coffee, multiple constituents (such as caffeine, polyphenols, or heterocyclic compounds) have anti-inflammatory and anti-oxidant properties, measured by reduced CRP levels and a lower risk of mortality from inflammatory diseases in regular coffee drinkers [9,41,42]. Chronic inflammatory processes, on the other hand, increase the risk for vascular dementia, Alzheimer’s Disease, and stroke, and are associated with an increased WMH prevalence at baseline, an increased WMH progression over time, and a reduced white matter integrity 20 years later [43,44,45,46,47]. The negative relationship between coffee consumption, vascular brain damage and cognitive decline can therefore be explained by the anti-inflammatory properties of coffee [3,4]. Only one previous study analyzed the microstructural integrity in the context of coffee [24]. They found that a moderate-to-high coffee consumption—reflecting approximately 1–6 cups of coffee per day—was associated with better microstructural integrity in 145 elderly individuals [24]. The results are comparable to our findings, including the more sensitive marker PSMD, with a lower PSMD in middle-aged to elderly individuals with a moderate coffee consumption of 1–4 cups per day.

Next to its anti-inflammatory mechanisms, coffee consumption has an influence on the central nervous system through its binding to adenosine receptors. The constituent caffeine is highly soluble in lipid and water, which allows caffeine to easily cross the blood-brain barrier. Caffeine has a chemically similar structure to adenosine and can, once in the brain, act as an adenosine receptor antagonist by binding to A1 and A2a receptors [2]. Via blockage of adenosine receptor A2a, caffeine can reduce amyloid-β induced toxicity [10]. In humans, coffee consumption was associated with a lower amyloid-β positivity on PET scans and, subsequently, a lower risk of dementia [4,22]. A midlife coffee consumption of 3–5 cups per day was associated with a 70% lower risk of incident dementia [3]. Cortical thickness as an MR imaging marker of neurodegeneration was higher in individuals with a coffee consumption of 3–4 cups per day in our cohort. Only one previous study considered cortical thickness in predefined regions of interest in the context of coffee consumption [22]. Previous results measuring cortical thickness indirectly via grey matter volume are heterogeneous with either positive, negative, or no associations of coffee consumption [21,25,26,48]. Moreover, adenosine receptor antagonists were protective against hippocampal damage after induced neurotoxicity and brain damage after ischemic stroke, most probably due to the suppression of glutamate release [49,50]. Similar mechanisms are suggested for the neuroprotective associations of caffeine, e.g., against the depletion of striatal dopamine levels in a Parkinson’s Disease mice model [51]. In humans, a daily coffee consumption of 3 cups was associated with a lower risk of Parkinson’s Disease in a large meta-analysis including 901,764 participants, and associated with 20% lower risk of incident stroke [8,52,53].

Coffee consumption may also be neuroprotective in an indirect way by reducing cardiovascular risk factors such as diabetes or metabolic syndrome [11,12]. A large umbrella review including 218 meta-analyses revealed that the reduced risk of diabetes mellitus is one of the most beneficial outcomes of regular coffee consumption [1]. Since cardiovascular risk factors are the major cause in the development of CSVD, coffee might help in reducing the degree of CSVD in individuals with high cardiovascular risk [54]. Although our study is limited in its cross-sectional design, we observed that the prevalence of diabetes mellitus was 13.96% in participants drinking less than 1 cup of coffee per day, compared to a prevalence of 5.33% in participants consuming more than 6 cups of coffee per day (Table 1).

A U-shaped relationship between coffee consumption and health outcomes has been reported in previous studies, with a moderate coffee consumption found in individuals with the most beneficial health parameters. Drinking 2.5–5 cups of coffee per day was associated with a reduced risk of dementia and metabolic syndrome, and a consumption of 1–3 cups per day was associated with a reduced risk for stroke, cardiovascular diseases, coronary heart disease, Parkinson’s Disease, and overall mortality [3,8,11,41,52]. Our findings also indicate a U-shaped association between microstructural white matter integrity, cortical thickness, and coffee consumption with better associations on the structural MRI for a consumption of 1–4 cups per day, while individuals with a daily consumption of 5 or more cups did not significantly differ from individuals drinking <1 cup in any MRI parameter. It is suggested that coffee has both beneficial and detrimental associations with health, where the detrimental mechanisms of a very high coffee consumption outweighs the positive, serving a possible explanation for the U-shaped association of coffee with health parameters [52]. At the same time, the comparability of studies is limited due to differences in portion sizes leading to a high risk of bias and very low quality of evidence in meta-analyses [55].

This study has strengths and limitations. In a large middle-aged to elderly population, we examined the relationship between coffee consumption and several brain MRI parameters. We quantified PSMD as novel imaging marker of microstructural integrity, and additionally included measures of vascular brain damage and neurodegeneration to better understand the association of coffee consumption with structural brain alterations. The study included data from a large population-based cohort, and we applied an advanced processing protocol on the MR images. Still, only cross-sectional data are currently available, and no conclusions about causality can be drawn from observational, cross-sectional data. The lack of longitudinal data in our analysis should be considered a limitation. As observed in this cohort, coffee consumption was positively correlated with the consumption of alcohol and smoking, which are known to negatively influence both overall and neurological health. Therefore, the true strength of the association between coffee and health parameters might even be underestimated. At the same time, individuals consuming more coffee tended to be younger, higher educated, and less often diagnosed with hypertension or diabetes. Despite the inclusion of important covariates in the linear regression models, we cannot rule out potential residual confounding factors. This study was intended to be exploratory, given that multiple predictors were assessed and adjustment for multiple testing was not performed. In addition, we did not compare the association of caffeinated with decaffeinated coffee, because of the small sample size drinking exclusively decaffeinated coffee (N = 7). The suggested associations of caffeine on neurological health need to be studied in detail in larger cohorts.

## 5. Conclusions

To summarize, a coffee consumption of 1–4 cups per day is associated with a lower PSMD, indicating better microstructural integrity. Moreover, we found that a coffee consumption of 3–4 cups per day was associated with preserved cortical thickness. In total, a moderate coffee consumption was related to better structural brain parameters than a low coffee consumption in a population-based cohort with middle-aged to elderly individuals. Further research is necessary to determine whether coffee consumption has a potentially protective effect against microstructural brain alterations longitudinally.

## Figures and Tables

**Table 1:** Descriptive statistics of the overall cohort, as well as for each group of coffee consumption.

Characteristics		Coffee Consumption					*p*-Value *
	Overall Cohort	<1 c/d	1–2 c/d	3–4 c/d	5–6 c/d	>6 c/d	
Group size	**2316**	**459**	**1020**	**581**	**176**	**80**	
**Sociodemographic characteristics**							
Age, median (IQR)	65 (14)	67 (13)	67 (13)	62 (14)	61 (13)	60 (9)	**<0.001**
Female sex, n (%)	1026 (44.3)	191 (41.61)	501 (49.12)	254 (43.72)	55 (31.25)	25 (31.25)	**<0.001**
Education ^1^							**<0.001**
Low, n (%)	98 (4.23)	25 (5.75)	47 (4.78)	18 (3.2)	6 (3.49)	2 (2.53)	
Medium, n (%)	1087 (46.93)	219 (50.34)	472 (47.97)	275 (48.85)	80 (46.51)	41 (51.9)	
High, n (%)	1048 (45.25)	191 (43.91)	465 (47.26)	270 (47.96)	86 (50)	36 (45.57)	
**Cardiovascular risk factors**							
Smoking, n (%)	320 (13.82)	37 (9.3)	113 (12.58)	110 (21.07)	39 (24.68)	21 (28.77)	**<0.001**
Diabetes mellitus, n (%) ^2^	210 (9.07)	61 (13.96)	88 (9.23)	45 (8.43)	12 (7.14)	4 (5.33)	**0.01**
Hypertension, n (%) ^3^	1611 (69.56)	330 (73.99)	734 (74.37)	380 (68.1)	121 (69.94)	46 (61.33)	**0.015**
BMI, median (IQR)	26.15 (9.45)	26.18 (5.59)	25.96 (5.44)	26.11 (5.43)	26.49 (4.59)	28 (4.9)	**0.02**
Alcohol consumption [monthly frequency], median (IQR)	3 (9)	3 (9)	3 (7)	10 (13)	10 (14)	3 (9)	**0.001**
Adherence to the Mediterranean diet, median (IQR)	4 (3)	4 (3)	4 (3)	4 (3)	5 (3)	4 (2)	0.945
**MRI parameters**							
Brain volume [mL], median (IQR)	1210.3 (166.6)	1204.38 (157.79)	1195.82 (168.59)	1226.89 (162.09)	1238.15 (162.73)	1240.43 (150.83)	**<0.001**
Mean cortical thickness [mm], median (IQR)	2.61 (0.13)	2.6 (0.13)	2.61 (0.13)	2.62 (0.13)	2.61 (0.11)	2.62 (0.11)	**<0.001**
WMH volume [mL], median (IQR)	1.48 (2.22)	1.61 (2.18)	1.59 (2.51)	1.29 (2.09)	1.32 (1.93)	1.28 (1.71)	**0.002**
PSMD [×10^5^], median (IQR)	22.3 (4.31)	22.77 (4.61)	22.52 (4.36)	21.96 (4.05)	22.24 (4.37)	21.44 (2.81)	**<0.001**

All demographic variables were tested for significant difference between groups of coffee consumption. *p*-Values < 0.05 were interpreted as significant and are indicated in bold. * Pearson Chi-square test applied for categorical variables; Kruskal–Wallis test applied for continuous variables. ^1^ Educational level as defined by the International Standard Classification of Education (ISCED). ^2^ Prevalence of diabetes mellitus was determined based on fasting serum glucose level (>126 mg/dL), non-fasting serum glucose level (>200 mg/dL), or self-report. ^3^ Prevalence of hypertension was determined based on blood pressure (≥140/90 mmHg), intake of antihypertensive medication, or self-report. Abbreviations: BMI = body mass index, c/d = cups per day, IQR = interquartile range, mL = milliliter, mm = millimeter, MRI = magnetic resonance imaging, PSMD = peak width of skeletonized mean diffusivity, WMH = white matter hyperintensities.

**Table 2:** Results of the linear regression models examining the association between coffee consumption and microstructural brain parameters.

Dependent Variable	PSMD [×10^5^]		Mean Cortical Thickness [mm]		log WMH Load ^4^	
	**β**	***p***	**β**	***p***	**β**	***p***
Intercept	23.96	**<0.001**	2.571	**<0.001**	−6.967	**<0.001**
Age	1.917	**<0.001**	−0.025	**0.001**	0.558	**<0.001**
Sex—female	−0.84	**<0.001**	0.009	0.074	0.273	**<0.001**
Education—medium ^1^	−0.705	0.125	0.031	**0.012**	−0.148	0.257
Education—high ^1^	−0.663	0.155	0.044	**<0.001**	−0.294	**0.027**
Hypertension—yes ^2^	0.455	**0.029**	−0.006	0.245	0.133	**0.026**
Diabetes—yes ^3^	0.911	**0.005**	−0.013	0.147	0.097	0.299
Smoking—yes	0.577	**0.023**	−0.013	0.06	0.153	**0.035**
Alcohol consumption	0.303	**0.001**	−0.006	**0.017**	0.025	0.358
BMI	0.114	0.221	0.008	**0.002**	0.015	0.586
Mediterranean diet	−0.002	0.986	−0.001	0.569	−0.039	0.123
Coffee consumption—1–2 c/d	−0.542	**0.022**	0.004	0.544	−0.025	0.71
Coffee consumption—3–4 c/d	−0.591	**0.028**	0.018	**0.015**	0.061	0.429
Coffee consumption—5–6 c/d	−0.014	0.97	0.002	0.831	0.001	0.994
Coffee consumption—>6 c/d	−0.461	0.386	−0.001	0.941	−0.01	0.944

Three linear regression models were conducted with either PSMD, mean cortical thickness or log WMH load as the dependent variable. Coffee consumption was quantified in groups with <1 cup per day set as reference category. Numbers indicate coefficients and *p*-Values. The *p*-Values < 0.05 were interpreted as significant and are indicated in bold. ^1^ Educational level as defined by the International Standard Classification of Education (ISCED). Low educational level was set as reference. ^2^ Prevalence of hypertension was determined based on blood pressure (≥140/90 mmHg), intake of antihypertensive medication, or self-report. ^3^ Prevalence of diabetes mellitus was determined based on fasting serum glucose level (>126 mg/dL), non-fasting serum glucose level (>200 mg/dL), or self-report. ^4^ Log WMH load refers to the logarithm of WMH volume and normalization for estimated total intracranial volume. Abbreviations: β = standardized coefficient, BMI = body mass index, c/d = cups per day, log = logarithmic, mm = millimeter, *p* = *p*-Value, PSMD = peak width of skeletonized mean diffusivity, WMH = white matter hyperintensities.

## Data Availability

The data included in this article cannot be shared publicly for the privacy of individuals that participated in this study.
